# Boxer's knuckle: Sonographic anatomy and assessment of sagittal band tears of the dorsal hood

**DOI:** 10.1002/ajum.12363

**Published:** 2023-10-12

**Authors:** Michelle Fenech

**Affiliations:** ^1^ College of Clinical Sciences, Health, Medical and Applied Sciences, CQUniversity Brisbane Queensland Australia; ^2^ Department of Medical Imaging Royal Brisbane and Women's Hospital Brisbane Queensland Australia

**Keywords:** Boxer's knuckle, dorsal hood, extensor tendons, metacarpophalangeal joint, musculoskeletal ultrasound, sagittal bands, sonography

## Introduction

Hand injuries are common in amateur and professional boxers and result in time lost from training and competition.^1^, ^2^, ^3^ Injuries to the dorsal hood account for 16% of all hand and wrist injuries in boxers.^1^, ^3^ ‘Boxer's knuckle’ describes a closed injury to the metacarpophalangeal joint (MCPJ) of the hand and is used synonymously to describe tears of the sagittal bands of the dorsal hood and associated extensor tendon instability.^4^, ^5^ It can result from a direct blow to the flexed MCPJ, commonly from boxing or punching, or from relatively low‐energy repetitive injuries.^5^ Patients typically present with a painful and swollen dorsal MCPJ, and the space between knuckles, with pain associated with forming a closed fist, loss of full extension and snapping of extensor tendons with MCPJ flexion.^6^, ^7^ Boxer's knuckle soft tissue injuries are less appreciated than boxer's fracture that typically involves a fracture of the fifth or fourth metacarpal neck with volar angulation and can occur from a similar mechanism of injury.^8^

Tears of the sagittal bands of the dorsal hood can be clinically overlooked or underappreciated, as the symptoms can often be non‐specific, and the associated tendon subluxation or dislocation may not always be observed.^9^, ^10^ If not diagnosed and treated adequately and in a timely manner, sagittal band tears can result in long‐term persistent pain at the MCPJ and hand function impairment.^11^, ^12^, ^13^ Diagnostic imaging, including sonography, can play an important role in directly imaging the soft tissue structures surrounding the MCPJ and diagnosing sagittal bands tears and tendon instability; however, an appreciation of the mechanism of injury, sonographic anatomy, sonographic technique, and normal and abnormal sonographic appearances is required.

## Sonographic anatomy of the extensor mechanism of the metacarpophalangeal joints of digits 2–5

The anatomy of the extensor (dorsal) mechanism of digits 2–5 of the hand is complex and often overwhelming. It combines an array of dorsal soft tissue structures including extensor tendons, the dorsal plate and the dorsal hood (extensor expansion).^14^ The dorsal hood is interrelated with intermetacarpal and palmar hand structures which aid in producing finger movement and MCPJ stability.^15^ The intermetacarpal structures include collateral ligaments, lumbrical and interosseous muscles and their associated tendons. Palmar structures of the hand around the MCPJ include the palmar plate, A1 pulley, flexor tendons, the deep transverse metacarpal ligament (DTMCL) and the associated neurovascular structures.^15^ The dorsal, intermetacarpal and palmar structures surrounding the MCPJ all need to be sonographically assessed in cases of suspected sagittal band tears.

## Dorsal tendons

**Figure 1:**
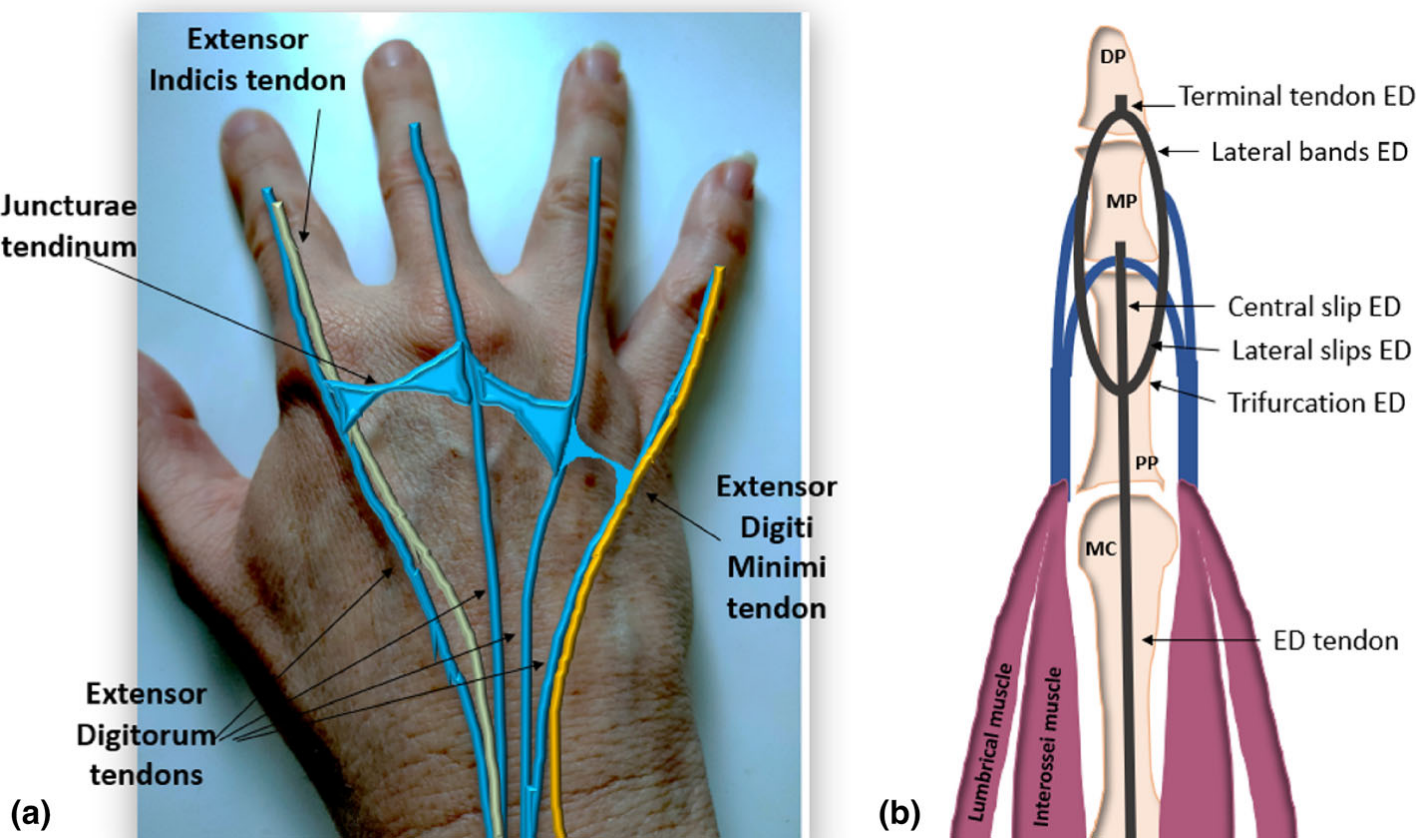
Anatomy of extensor tendons of the fingers. (a) Surface anatomy and relative location of the extensor tendons, which include the ED and the extensor indicis proprius tendon (to the second finger) and extensor digiti minimi tendon (to the fifth finger). (b) Anatomic diagram of the ED tendon with its trifurcation into a central slip and two lateral slips which join with tendons from the lumbrical and interossei muscles to form lateral ED bands. DP, distal phalanx; ED, extensor digitorum; MC, metacarpal; MP, middle phalanx; PP, proximal phalanx.

Extension of the proximal interphalangeal joint (PIPJ) and distal interphalangeal joint (DIPJ) is achieved *via* a combination of extensor tendons and intrinsic muscles of the hand (lumbrical and interossei muscles). Extrinsic tendons at the MCPJ are formed by the extensor digitorum (ED) tendon to the fingers, the extensor indicis proprius (EIP) tendon to the second (index) finger and the extensor digiti minimi (EDM) tendon to the fifth (little) finger.^16^ These tendons arise from the muscles that originate from the lateral elbow (ED and EDM) and forearm (EIP) and pass through dorsal compartments 4 and 5 of the wrist to the hand (Figure 1).

### The extensor digitorum tendon

**Figure 2:**
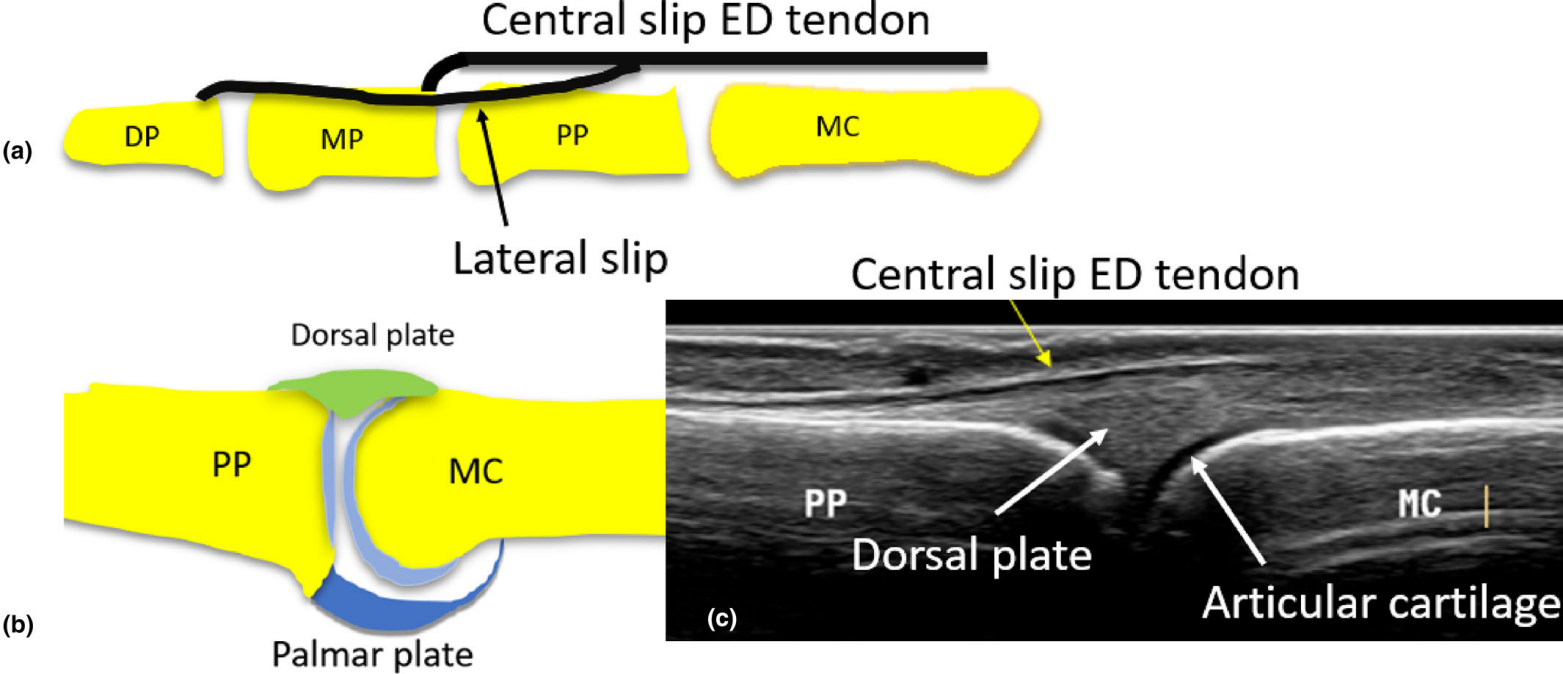
Anatomy of the ED tendon of the fingers. (a) Diagram demonstrating the tendon dorsal to the MC and MCPJ. It trifurcates into a central slip and two lateral slips distal to the MCPJ. The central slip inserts onto the dorsal aspect of the proximal aspect of the MP. The two lateral slips run dorsolaterally and insert *via* a terminal tendon onto the dorsal aspect of the DP. (b) Sagittal depiction of the MCPJ. The ends of bones of the MC and PP are lined with articular cartilage. The dorsal plate and palmar plates are present on the dorsal and palmar aspects of this joint. (c) Long‐axis sonographic image of the MCPJ demonstrating the ED tendon, dorsal plate and articular cartilage. DP, distal phalanx; ED, extensor digitorum; MC, metacarpal; MCPJ, metacarpophalyngeal joint; MP, middle phalanx; PP, proximal phalanx.

The ED tendon overlies the dorsal aspect of the MCPJs 2–5 and passes distally along the dorsal aspect of the fingers.^9^ It is centrally located over the dorsal aspect of the metacarpal (MC) heads (2–5). Sonographically, it appears oval in cross section at the level of the MCPJ and more flattened in cross section distal to the MCPJ. In long axis, the ED tendon sonographically appears as a thin fibrillar structure presenting immediately dorsal to the fibrocartilaginous dorsal plate of the MCPJ (Figure 2).

**Figure 3:**
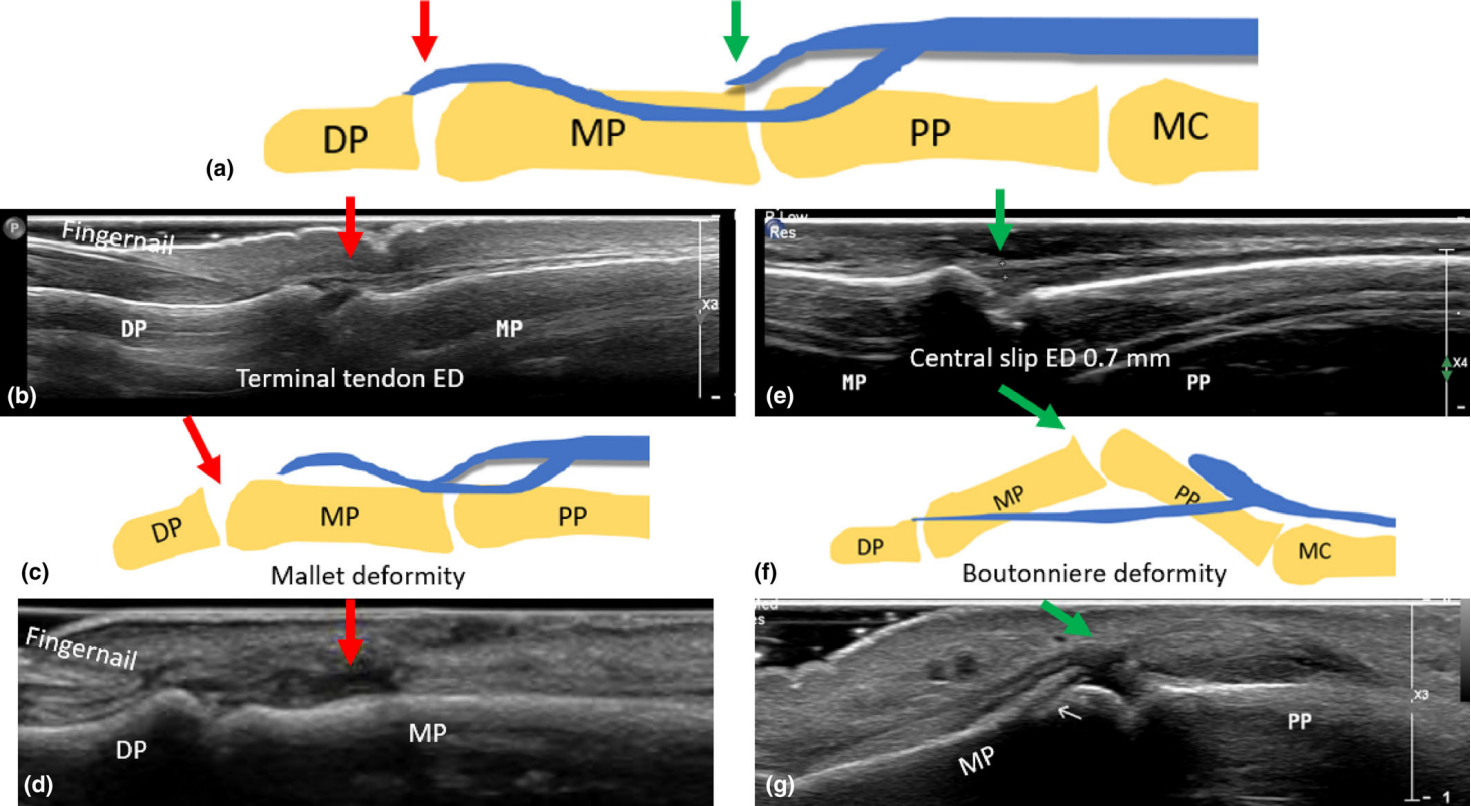
Distal components of the ED tendon. (a) Diagram with red arrow indicating the terminal tendon, and the green arrow indicating the central slip. (b) Long‐axis sonographic image of an intact ED terminal tendon onto the DP. (c) Diagram of torn terminal tendon (red arrow) and associated retraction of the lateral slip, which appears wavy (results in mallet finger deformity). (d) Sonographic demonstration of disrupted terminal tendon of ED with no avulsion. (e) Long‐axis sonographic image of intact central slip of ED (green arrow) onto the MP. (f) Diagram of torn central slip of ED (not extending to the MP) whilst the lateral slips remain intact (results in Boutonniere deformity). (g) Sonographic image of a torn central slip of ED tendon, which has retracted proximally. DP, distal phalanx; ED, extensor digitorum; MC, metacarpal; MCPJ, metacarpophalyngeal joint; MP, middle phalanx; PP, proximal phalanx.

At the level of the dorsal proximal phalanx, at variable distances distal to the MCPJ, the ED tendon trifurcates into a single central slip and two lateral slips.^5^ The central slip of the ED tendon extends along the midline dorsal aspect of the finger to insert onto the dorsal base of the MP.^17^ Complete tears to the central slip of the ED tendon can occur with blunt trauma, forced PIPJ flexion or PIPJ dislocation, and may be associated with an avulsion fracture of the dorsal base of the MP. When the central slip is completely torn in isolation, PIPJ extension will be affected, but lack of active PIPJ extension can be sometimes difficult to appreciate on physical inspection. The patient can present with a ‘Boutonniere deformity’, which occurs due to PIPJ flexion unopposed: the PIPJ appears flexed (as unable to extend the PIPJ due to central slip tendon disruption) and the DIPJ appears extended (Figure 3).

The two lateral (radial and ulnar) slips of the ED tendon blend with lateral bands from the lumbrical and interossei tendons to form conjoint lateral bands (see Figure 1b). These lateral bands course distally down the dorso‐lateral aspect of digits 2–5 and converge together to form a single terminal extensor tendon that inserts onto the dorsal base of the distal phalanx.^15^ Disruption to the terminal ED tendon, at the distal insertion onto the dorsal aspect of the distal phalanx base, can occur due to laceration or closed injuries. Forced flexion of the DIPJ when the joint is actively extended or a direct blow to the fingertip (commonly encountered in ball sports) can disrupt the terminal ED tendon. Tears to the terminal ED tendon can be associated with an avulsion fracture of the dorsal base of the distal phalanx. Patients may present with a ‘mallet finger’ deformity when the terminal ED tendon is completely torn, where they can extend their PIPJ, but are unable to extend their DIPJ, as the tendon is no longer attaching to the distal phalanx (Figure 3).

### Extensor indicis proprius and extensor digiti minimi tendons

**Figure 4:**
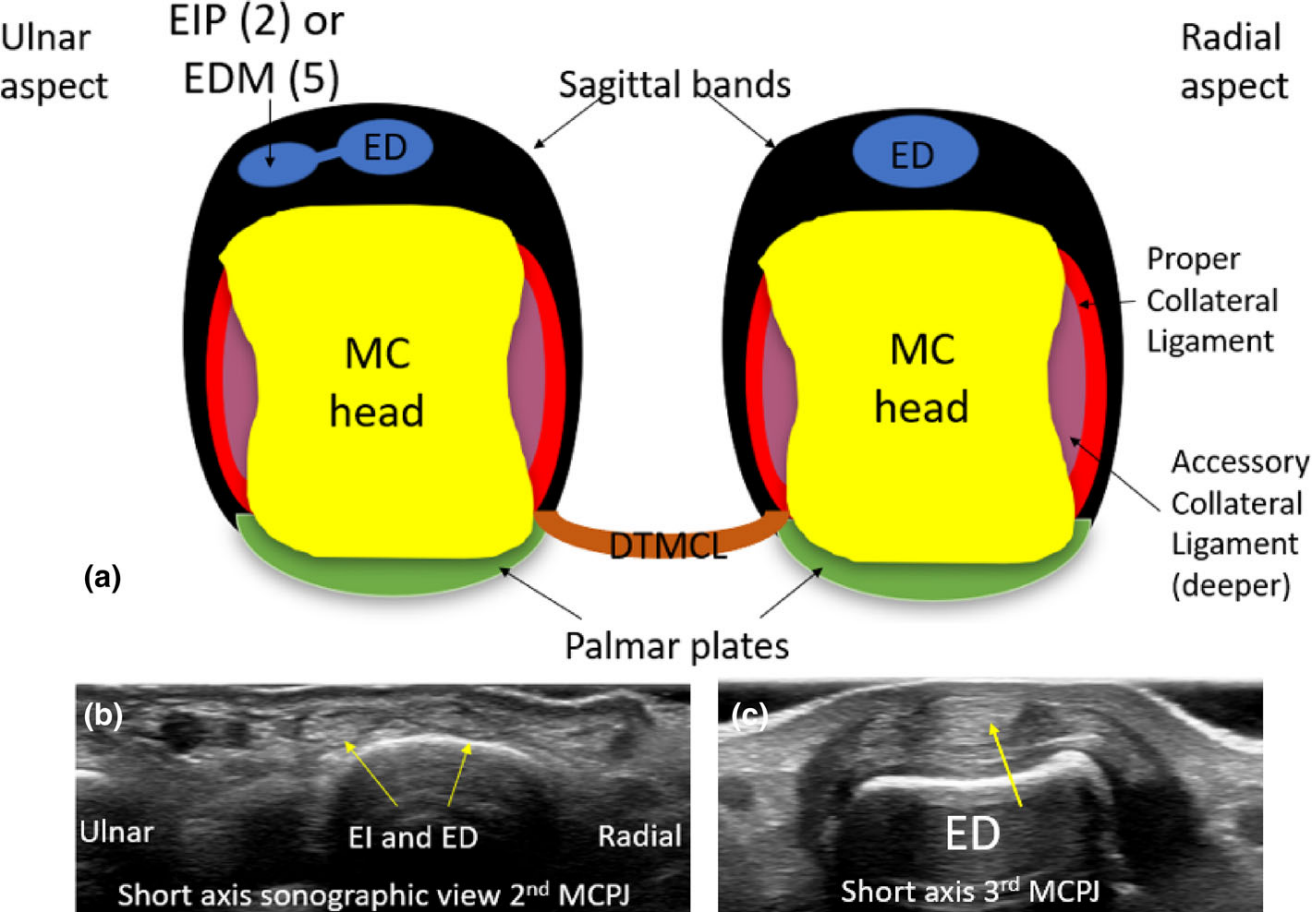
Short‐axis diagram (a) and sonographic images (b,c) of the MC head region of the hand. The ulnar relationship of the EIP or EDM tendons when present (on dorsal aspects) in digit 2 or 5 are demonstrated relative to the ED tendon. An interconnecting band between two tendons (in the second or fifth finger) is shown. In digits 3 and 4, a single ED tendon is present. (b) Short‐axis sonographic image of the dorsal MCPJ. Two extensor tendons (ED and EI) are seen within the sagittal bands overlying the second MCPJ. (c) A single ED tendon overlies the dorsal aspect of the third MCPJ (and the fourth MCPJ will have the same appearance). DTMCL, deep transverse metacarpal ligament; ED, extensor digitorum; EDM, extensor digiti minimi; EIP, extensor indicis proprius; EI, extensor indicis; MC, metacarpal; MCPJ, metacarpophalyngeal joint.

There is considerable variability reported in the anatomy of the extensor tendons of the hand, particularly in relation to extra extensor tendons which extend to digits 2 and 5. Generally, at the second and fifth MCPJs, two extensor tendons are usually present overlying the dorsal aspect: the EIP and ED tendons at the second MCPJ, and the EDM and ED tendons at the fifth MCPJ. The EIP and EDM tendons are present at the ulnar aspect of the ED tendons.^18^ Fine fibrous connections or slips exist between the ED and additional tendons to aid in maintaining their position^15^, ^19^ (Figure 4).

The EIP tendon (also referred to as the extensor indicis) runs through the fourth extensor compartment of the dorsal wrist along with the ED tendons. In the dorsal hand, the EIP tendon is present at the ulnar side of the ED tendon and overlies the dorsal aspect of the second MC and MC head, although variants where it has been observed to be present to the radial aspect of the ED have been reported.^18^ Unlike the ED tendon, the EIP tendon does not insert distally onto bone, but rather inserts variably onto the ulnar aspect of the dorsal hood of the second finger, at the proximal phalanx (PP) level.^9^ Sonographically, the EIP tendon can be identified at the level of the second MC head over its dorsal aspect, encapsulated by the sagittal band. Distal to the region of the sagittal band, the EIP tendon can be difficult to sonographically define as it blends with the transverse band of the dorsal hood.

The EDM tendon is also referred to as the extensor digiti quinti minimi (EDQM). The distal EDM tendon runs through the fifth extensor (dorsal) compartment of the wrist and travels distally over the dorsal aspect of the fifth MCPJ and inserts onto the ulnar aspect of the dorsal hood (transverse band) at the PP level.^20^ Sometimes, an ED tendon cannot be seen extending to the fifth finger, and it may be replaced with two EDM tendons.^20^ The presence and relative anatomy of the EIP and EDM tendons and their sonographic appearances must be appreciated when imaging the sagittal bands, to avoid confusion with longitudinal splits of the ED tendon.

### Juncturae tendini

Just proximal to the dorsal MCPJ 2–5, ED tendons are interconnected by juncturae tendini (juncturae tendinum = singular). The juncturae tendini consist of bands of connective tissue that extend transversely and obliquely between the extensor tendons in the second, third and fourth dorsal intermetacarpal spaces, linking the motion of ED tendons and contributing to their stability.^15^, ^16^ The juncturae tendini also redistribute force of the ED tendons, reduce excessive load of the sagittal bands and assist in stabilising the MCPJs.^16^ As juncturae tendini can be injured in hand injuries, their presence and integrity must be sonographically appreciated.

## The dorsal hood

**Figure 5:**
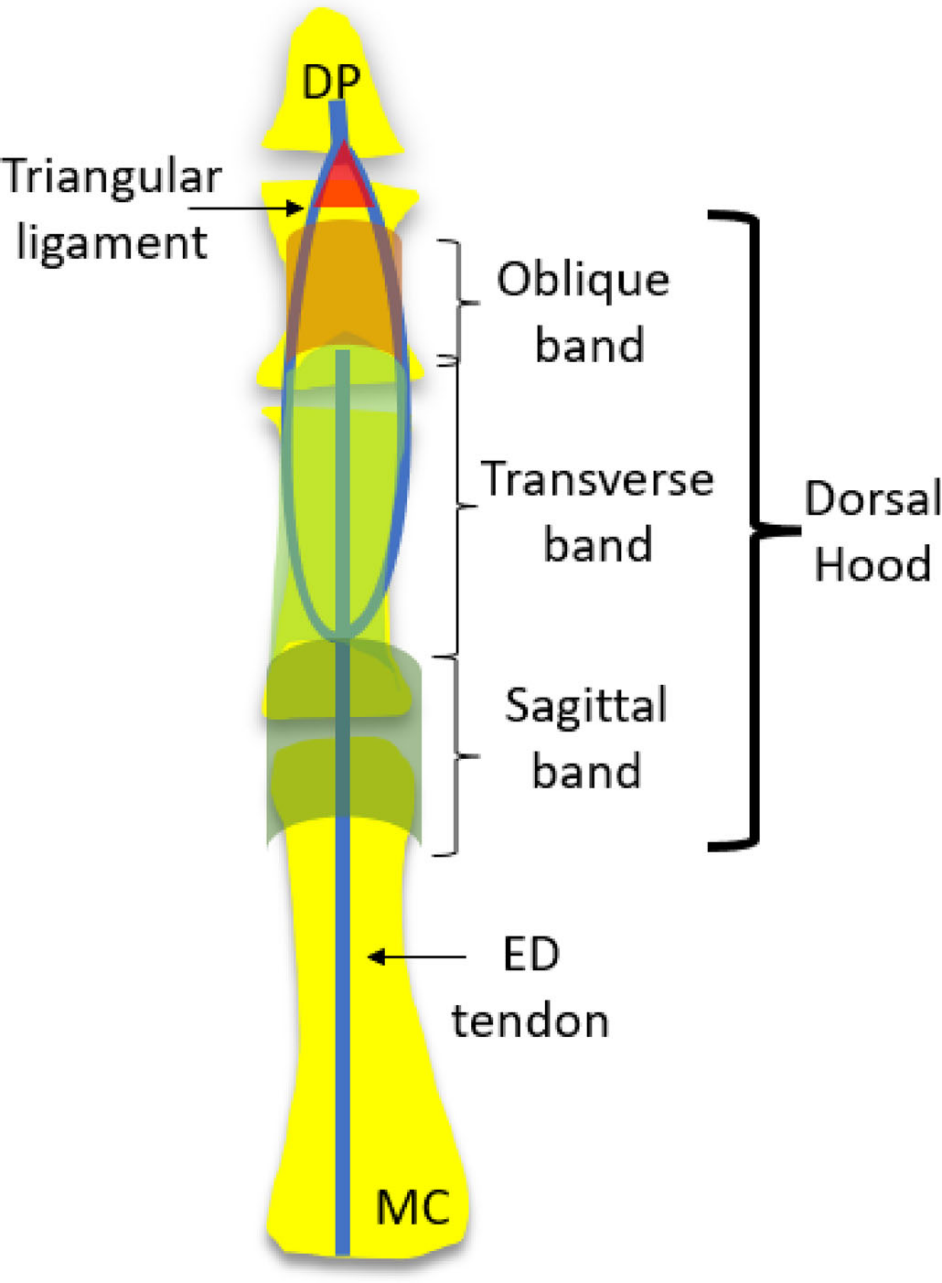
Diagram of relative locations of three retinacular components of the dorsal hood of digits 2–5 of the hand. The sagittal, transverse and oblique bands present in a proximal to distal orientation. The triangular ligament is not considered part of the dorsal hood, but rather is located distal to the dorsal hood. DP, distal phalanx; ED, extensor digitorum; MC, metacarpal.

The dorsal hood (also called the dorsal expansion or dorsal extensor mechanism) is a complex retinacular system over the dorsal or extensor aspect of the hand and fingers which acts to stabilise extensor tendons at the dorsal aspect of the MCPJ, PP and middle phalanx (MP).^5^ It is a coalescence of the all the extensor components and contributes to a broad, flat and thin aponeurotic expansion that covers 50% of the dorsal finger. It consists of three principle retinacular and stabilising bands from proximal to distal: sagittal, transverse and oblique bands^5^, ^15^ (Figure 5).

### Sagittal bands of the dorsal hood

**Figure 6:**
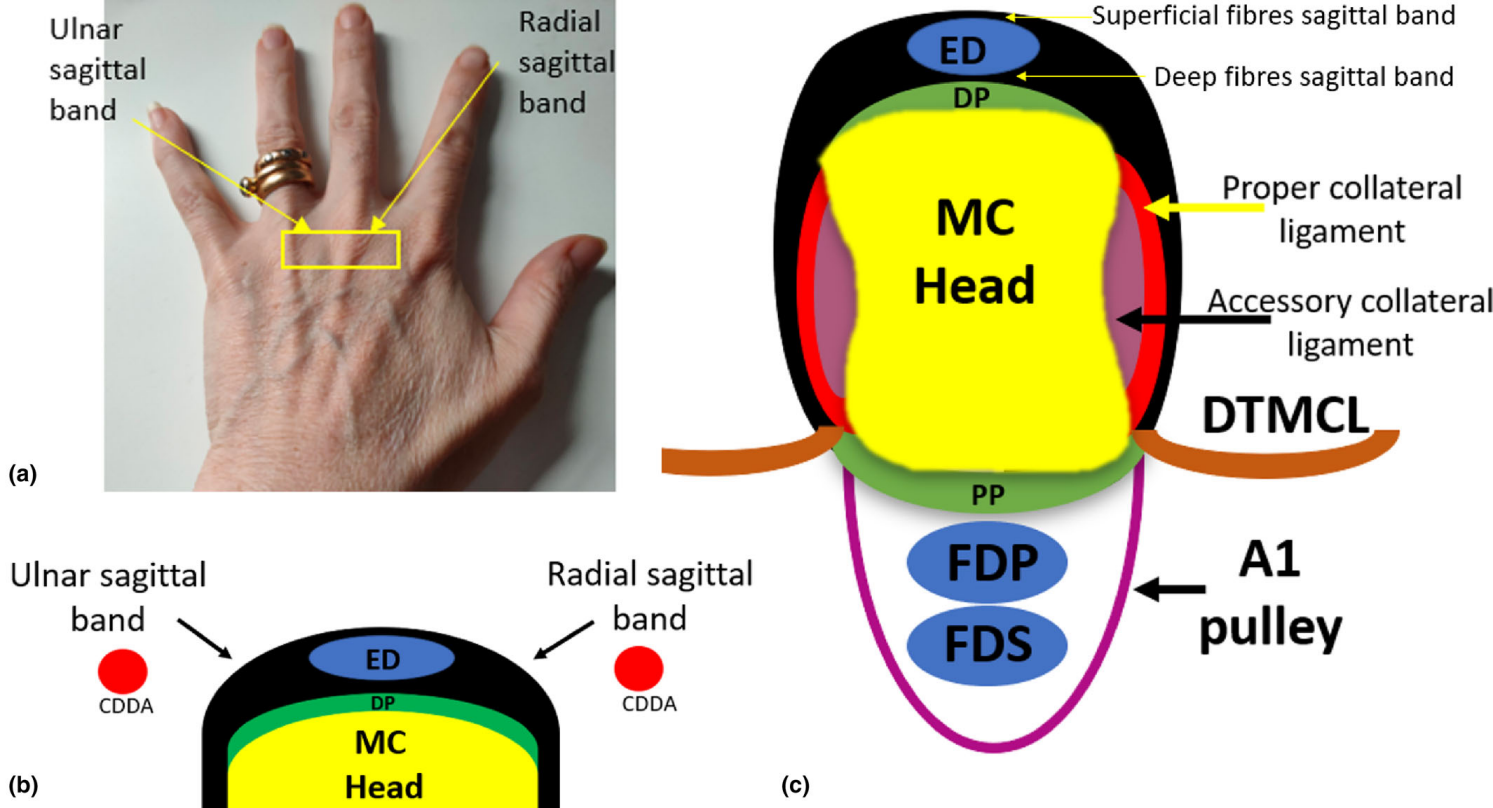
Sagittal bands of digits 2–5. (a) Location of the MCPJ and area for short‐axis sonographic imaging of this structure at the third MCPJ. (b) The sagittal bands (black) surround the ED tendon, and other structures surround the dorsal and intermetacarpal aspects of the metacarpal head. The radial and ulnar aspects of the metacarpal head must be appreciated. The CDDA can be sonographically appreciated in the dorsal intermetacarpal spaces. (c) Structures that surround the MC head include palmar‐sided structures. CDDA, common dorsal digital arteries; DP, dorsal plate; DTMCL, deep transverse metacarpal ligament; ED, extensor digitorum; FDP, flexor digitorum profundus tendon; FDS, flexor digitorum superficialis tendon; MC, metacarpal; MCPJ, metacarpophalyngeal joint; PP, palmar plate.

The sagittal bands are the proximal and most important component of the dorsal hood. They are located at the level of the MCPJ and surround the dorsal and intermetacarpal components of the MC head.^17^ They function to maintain the central position of the extensor tendons at the dorsum of the MCPJ during joint flexion and extension and prevent excessive tendon excursion, bowstringing, subluxation or dislocation.^5^, ^14^ The sagittal bands are dynamic structures that run perpendicular to the long axis of the ED tendon.^5^, ^16^ Sagittal band fibres run circumferentially around the MCPJ before inserting onto the palmar plate and the DTMCL on the palmar aspect of the hand.^15^ They envelop the extensor tendons, forming a tunnel through which the ED tendon travels, and consist of two layers relative to the ED tendon: a thinner superficial layer and a thicker deeper layer.^5^, ^14^, ^21^ There are radial‐ and ulnar‐sided components of a sagittal band, which continue around the MCPJ superficial to the radial and ulnar collateral ligaments^5^ (Figure 6).

### Transverse and oblique bands of the dorsal hood

Other fibrous structures of the dorsal hood include the transverse and oblique bands that are present distal to the sagittal bands and also serve to stabilise the extensor tendons.^5^ There is a paucity of literature reporting the detailed relative anatomy, sonographic appearances and injuries to these structures.^22^ The transverse band of the dorsal hood is also called the transverse retinacular ligament, and there is lack of consensus regarding the true extent of this ligament. It is variably described as extending distally from the distal edge of the sagittal bands (at the level of the trifurcation of the extensor tendons overlying the PP) to the proximal dorsal aspect of the MP.^15^, ^16^, ^19^ It covers the dorsal aspect of the PIPJ and insertion of the central slip of the ED tendon and envelops and stabilises it during PIPJ movement.^9^ The transverse band also prevents excessive dorsal migration of the lateral bands at the PIPJ.^16^

The oblique band of the dorsal hood is also called the oblique retinacular ligament or oblique ligament of Landsmeer.^19^ It overlies and assists to stabilise the lateral extensor tendon slips and bands at the MP level.^15^ The fibres of the oblique band are more compact and run at an angle of 30 degrees relative to the extensor tendon.^15^ The oblique band is sonographically inseparable from the transverse band and links the motion between the interphalangeal joints.^15^, ^16^ Isolated injuries to the transverse and oblique bands, such as in lacerations, have not been associated with extensor tendon instability.^15^

There is also a distally located triangular ligament, although it is not formally considered part of the dorsal hood.^15^ It overlies the dorsal DIPJ and connects the distal portions of the lateral bands of the ED tendon before they join to form the terminal tendon.^19^ It prevents the lateral movement of the distal lateral bands of the ED tendon during finger flexion.

## Further structures surrounding the metacarpophalangeal joints

The sagittal bands are inter‐related with palmar structures of the MCPJ, which include the palmar plates, the DTMCL, collateral ligaments and intrinsic muscles (lumbricals and interossei). As concurrent injuries may occur to these structures, they should also be sonographically assessed when sagittal band tears are suspected.

### Palmar plates

**Figure 7:**
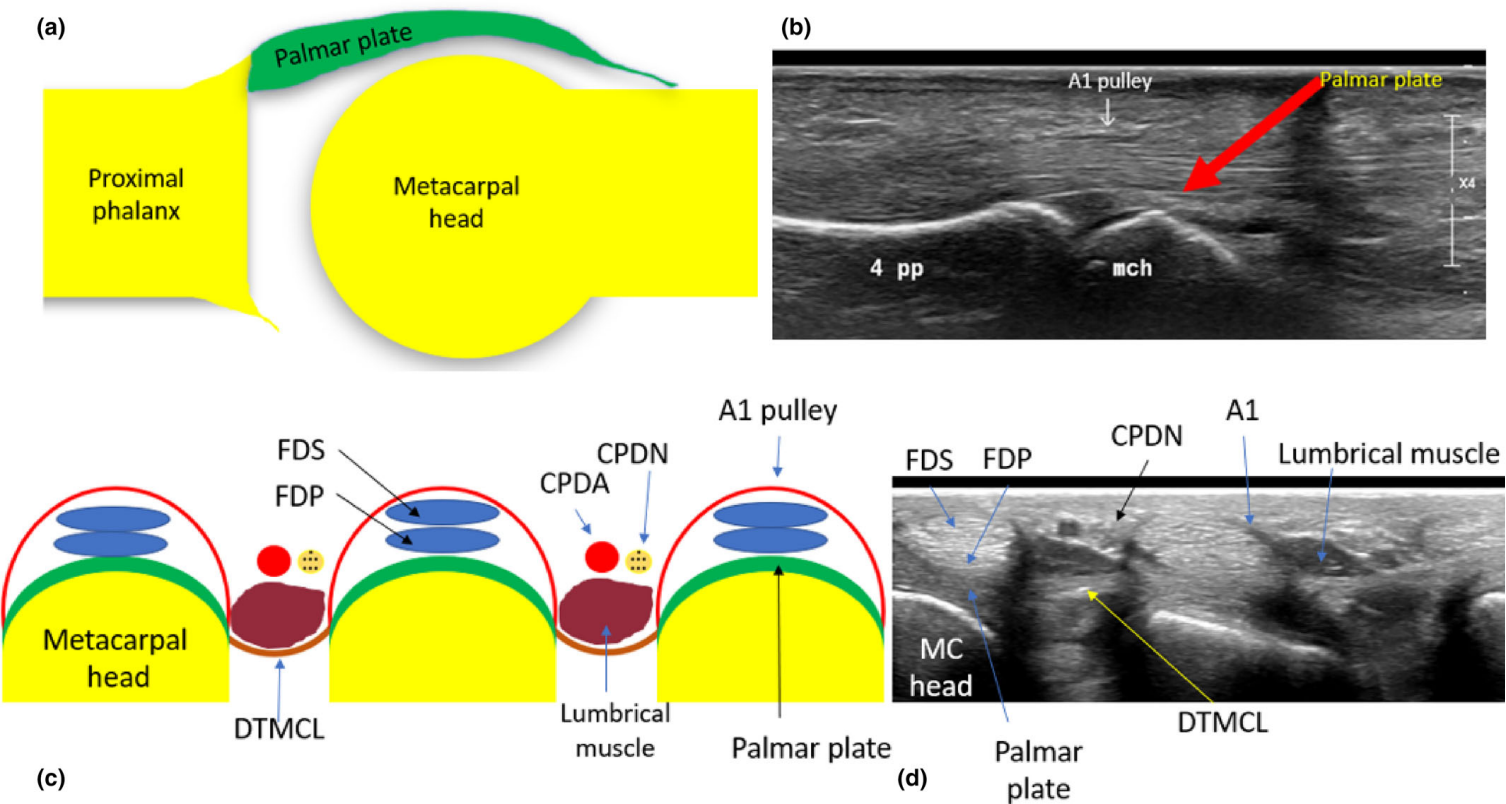
Anatomy of the palmar aspect of the metacarpophalangeal joints 2–5. (a) Long‐axis diagram demonstrating the palmar plate. (b) Long‐axis sonographic image demonstrating the mch and its hypoechoic overlying articular cartilage, fourth pp, the palmar plate and overlying FDS and FDP tendons and the overlying A1 pulley. (c) Short‐axis diagram of the palmar aspect of the metacarpal heads of the hand and associated structures including the DTMCL, palmar plates, flexor tendons, A1 pulley, lumbrical muscles and CPDN and CPDA. (d) Associated short‐axis sonographic image of the palmar aspect of the metacarpal heads demonstrating the palmar plates and DTMCL. CPDA, common palmar digital artery; CPDN, common palmar digital nerve; DTMCL, deep transverse metacarpal ligament; FDP, flexor digitorum profundus tendon; FDS, flexor digitorum superficialis tendon; MC, metacarpal; mch, metacarpal head; pp, proximal phalanx.

The palmar (volar or glenoid) plates are located at the palmar aspect of the MCPJ as well as the interphalangeal joints and act to reinforce the joint capsule and limit joint hyperextension.^23^ At the second to fifth MCPJs, the palmar plate is fibrocartilaginous at its broader distal insertion onto the palmar PP base and, at its proximal origin onto the MC neck, it becomes thin and membranous. It assists the sagittal band by pulling the ED tendon over the MC head with MCPJ extension.^16^ The palmar plates of digits 2–5 are connected transversely by the DTMCL. The flexor tendons of digits 2–5 (flexor digitorum superficialis and flexor digitorum profundus) pass through the A1 annular pulley, which is connected to the palmar plate (Figure 7).

### The deep transverse metacarpal ligament

The DTMCL can be an underappreciated structure. It connects the palmar plates of the MCPJ of digits 2–5 in the transverse plane and maintains the transverse metacarpal arch.^24^ It prevents the MC heads from splaying apart and aids the grip strength of the hand. It also merges with the accessory collateral ligaments.^24^ Although tears to the DTMCL are reported as rare, they can occur with forceful or blunt trauma, and concurrently with metacarpal fractures, MCPJ collateral ligament tears and sagittal band tears.^25^

### Collateral ligaments of the metacarpophalangeal joints

The collateral ligaments consist of both, proper and accessory ligaments.^26^ The MCPJ ligaments are present deep to the sagittal band and stabilise the joint on the radial and ulnar aspects. Injuries to the collateral ligaments can occur anywhere along their length. They are best referred to as the radial or ulnar collateral ligaments rather than medial and lateral ligaments, as it can be difficult to identify lateral and medial aspects of the hand correctly when rotating the hand from a pronated to supinated position during sonographic assessment. Appreciating their relative anatomic orientation and positioning relative to the sagittal bands is important for sonographic assessment, as partial tears to the collateral ligaments can frequently occur in association with sagittal band tears.^27^ When partially torn, the collateral ligaments appear to be sonographically thickened and hypoechoic.

**Figure 8:**
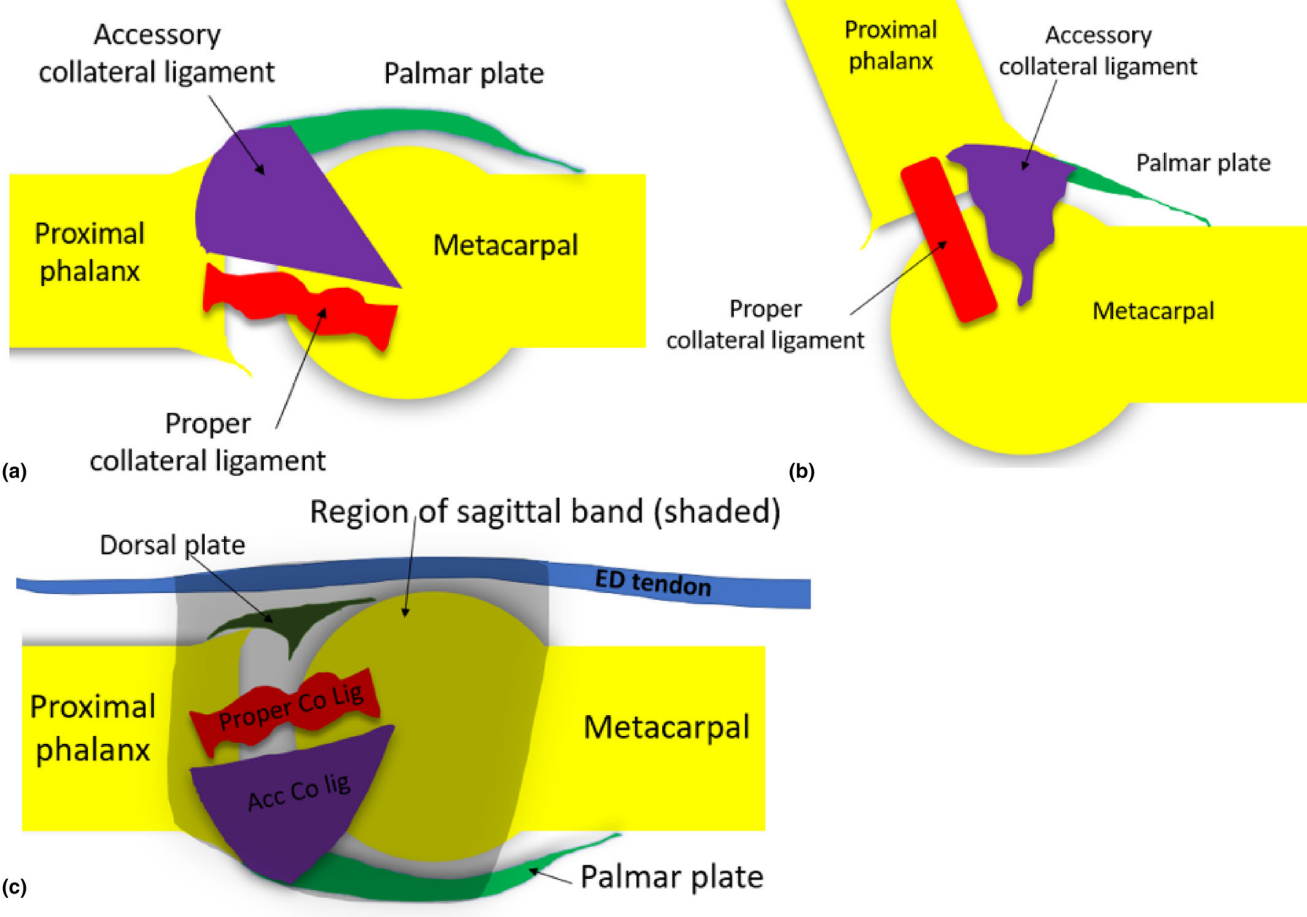
Collateral ligaments of the MCPJ of digits 2–5. (a) When the MCPJ is extended, the accessory collateral ligament is taut, and the proper collateral ligament is lax (appears wavy in diagram). (b) When the MCPJ is flexed, the proper collateral ligament becomes taut, and the accessory collateral ligament becomes lax. (c) The sagittal band (transparent in image) overlies both the accessory and proper collateral ligaments. The accessory collateral ligament is more palmar in location relative to the proper ligament. Acc Co Lig, accessory collateral ligament; Co Lig, collateral ligament; ED, extensor digitorum; MCPJ, metacarpophalyngeal joint.

The proper collateral ligament arises from the MC head more dorsally and inserts onto the base of the proximal phalanx, distal to the articular cartilage.^28^ They become taut when the joint is flexed.^29^ The accessory collateral ligament arises just proximal to the proper collateral ligament on the MC, and fans out at its distal and palmar aspect, to insert broadly onto the palmar plate near its proximal phalanx insertion.^22^ Accessory collateral ligaments become lax as the MCPJ is flexed^30^ (Figure 8).

### Lumbrical and interossei muscles

The intrinsic muscles of the hand consist of the lumbrical and interossei muscles, which contribute to the dorsal hood and the ED tendons. The distal tendons of these muscles merge and join the lateral slips of the ED tendons to form the conjoint lateral bands of the distal ED tendons. The interossei muscles consist of dorsal (abductor) and palmar (adductor) interossei muscles, and they make a small contribution to the central slip of the ED tendon. The lumbrical and interossei muscles aid to flex the MCPJs, extend the interphalangeal joints and assist with maintaining MCPJ extension in digits 2–5.^16^ The lumbrical muscles are more palmar in location and are present at the palmar aspect of the DTMCL. The interossei muscles and tendons can be sonographically differentiated from the lumbrical muscles as they are present at the dorsal aspect of the DTMCL.

## Sonographic assessment of sagittal bands and normal sonographic appearances

To sonographically assess the sagittal bands of the dorsal hood, a high‐frequency (≥12 MHz) linear transducer is required. The dorsal aspect of the MCPJs and the sagittal bands should be assessed both with fingers extended, and with the hand in a fist formation with dynamic imaging during flexion and extension required. To allow scanning of the dorsal hand during flexion and extension of the MCJP, the hand can be placed over the edge of a foam pad, rolled up face washer or gel bottle. Due to the bony nature of the dorsal MCPJ, sufficient gel is required to ensure transducer contact is maintained during dynamic imaging with flexion and extension. A hockey stick transducer, with a small footprint can facilitate better transducer contact with dynamic imaging. Transducer pressure must also be light enough to allow extensor tendon subluxation or dislocation to be demonstrated in real time, as greater transducer pressure may prevent or obscure tendon movement.

### Short‐axis imaging of the MCPJ and the sagittal bands

**Figure 9:**
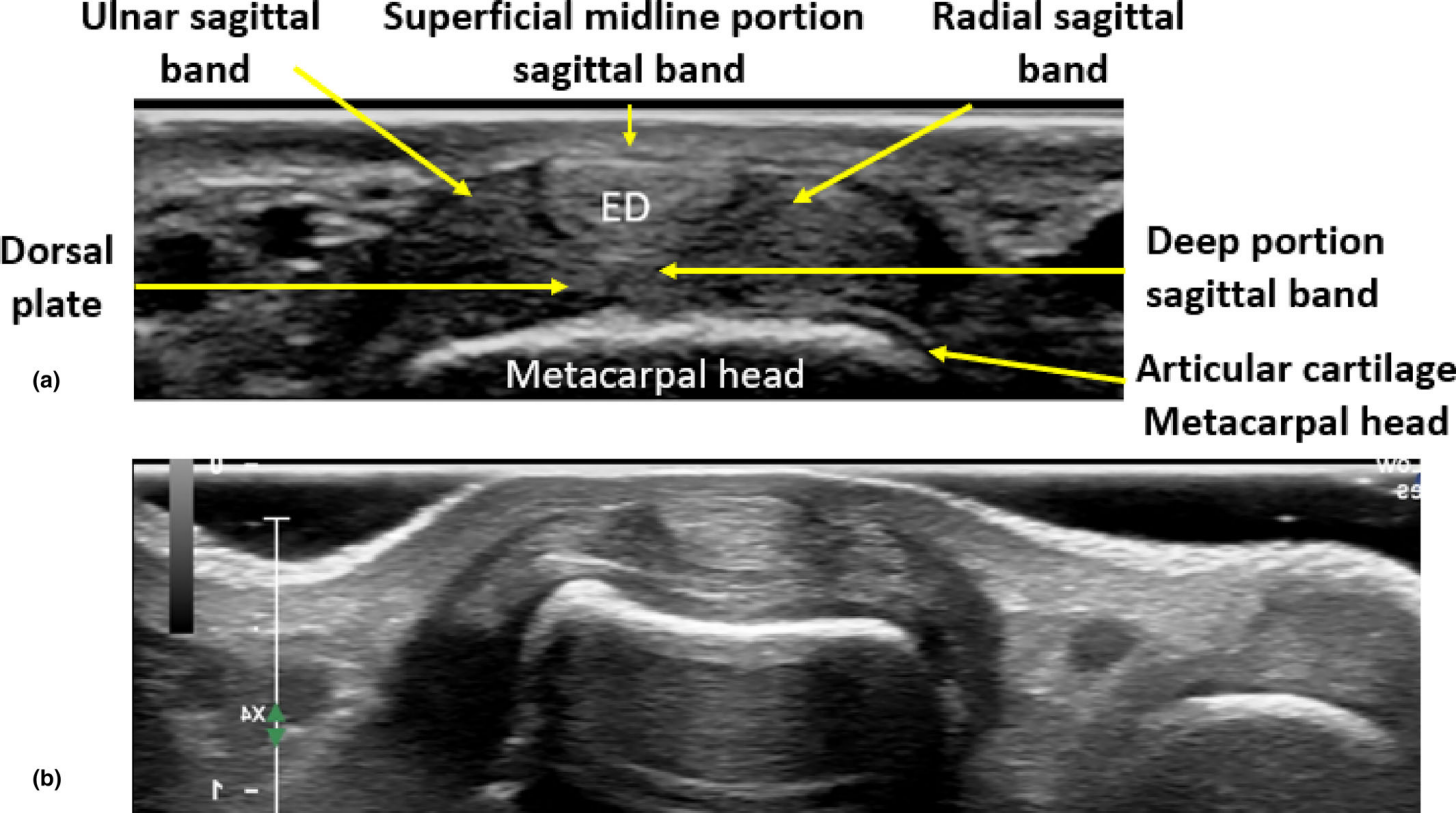
Short‐axis sonographic image of the dorsal aspect of the uninjured third MCPJ with (a) and without (b) labels. The ED tendon is demonstrated in short axis. The superficial and deep portions of the sagittal band in midline are seen with the deep portion superficial to the dorsal plate and the articular cartilage of the metacarpal head. The radial and ulnar portions of the sagittal band are demonstrated. ED, extensor digitorum; MCPJ, metacarpophalyngeal joint.

Short‐axis (transverse) imaging of the MCPJ will allow numbering of the MCPJ of interest, relative to adjacent joints, and the orientation of the radial and ulnar sagittal bands of the joint of interest. When imaging the dorsal aspect of the MCPJ in short axis, uninjured sagittal bands will appear homogenous in echotexture, extending both superficial and deep, and to the radial and ulnar aspects of the extensor tendons.^9^ Multiple degrees of angulation of the transducer are required as the sagittal bands are anisotropic structures and can change in echogenicity with altering angles of insonation (Figure 9).

The thickness (depth) of uninjured sagittal bands, measured from the short‐axis plane of the MCPJ, has been variably reported to measure from 0.42 to 0.72 mm with no thickness difference identified between digits 2 and 5 in dominant and non‐dominant limbs.^5^, ^21^ Comparison between adjacent asymptomatic MCPJs and the contralateral limb is important particularly when partial sagittal band tears are suspected, where the sagittal band can become thickened. The degree of MCPJ flexion and extension should be noted when imaging the sagittal bands and extensor tendons and comparing thickness to other MCPJs.

Using short‐axis imaging of the MCPJ, the sagittal bands should be sonographically assessed throughly: from the proximal dorsal MCPJ to mid‐PP level. Dynamic assessment should be performed to look for real‐time positioning of ED tendon relative to the dorsal aspect of the MC head. This can also allow for the identification of subluxation/dislocation of the extensor tendons to either the radial or ulnar aspects of the MC head associated with a sagittal band tear. The palmar extensions of the sagittal bands should be assessed. The sagittal bands superficial to the collateral ligaments in the intermetacarpal space can be difficult to demonstrate sonographically due to transducer access to this region, where they are oriented parallel to the incident sound.^15^

The attachment of the sagittal bands to the palmar plates and DTMCL should be identified by scanning the palmar aspect of the hand. The uninjured palmar plates should appear homogenous in echogenicity and echotexture. Hypoechoic gaps or defects of the palmar plate, particularly close to the proximal phalanx insertion can indicate palmar plate degeneration or tears (long‐axis and short‐axis imaging is required for palmar plate assessment). The DTMCL should be assessed sonographically in the transverse plane. When normal in appearance it should demonstrate an even echogenicity and thickness in multiple intermetacarpal spaces of the affected hand and contralateral hand. Dynamic imaging with abduction of the fingers can be used to assess for DTMCL integrity. The lumbrical muscles and common palmar digital nerves and vessels should be identified to the palmar aspect of the DTMCL. From the palmar aspect of the hand at the MCPJ levels, the interossei muscles will be identified dorsal (and deep) to the lumbrical muscles and the DTMCL.

### Long‐axis imaging of MCPJ

Long‐axis sonographic imaging of the dorsal aspect MCPJ is performed to assess the joint capsule, position of the extensor tendons and thickness of the sagittal bands. The triangular‐shaped dorsal plate should be identified as a homogenous structure when uninjured (see Figure 2c).^31^ When a moderate‐sized joint effusion is present, the dorsal plate may be elevated by fluid, relative to the articular cartilage overlying the MC head, and also appear blunted in shape.^31^ The unaffected ED tendon should be seen sonographically as a thin, continuous, fibrillar structure of similar thickness throughout, overlying the midline dorsal aspect of the MCPJ. Its distal extensions to the MP (central slip) and distal phalanx (combined terminal tendon of two lateral bands) should be demonstrated. The normal position of the ED tendon along the mid‐aspect of the joint should be maintained with dynamic imaging incorporating MCPJ extension and flexion when the sagittal bands are intact. The EIP and EDM tendons should also be assessed in long axis when examining the second and fifth MCPJs.

## Sonographic imaging of sagittal band tears

Traumatic tears to the sagittal bands result from direct trauma to the dorsum of the MCPJ or resisted joint extension. Sagittal band tears tend to occur as longitudinal splits, extending in a proximal‐to‐distal orientation, and can result in extensor tendon instability and possibly impaired MCPJ extension.^22^ Tears can involve the proximal and/or distal component of the sagittal band, and the extent of the tear should be defined.^5^ Sagittal band tears usually involve the third or fourth MCPJ.^19^ The third (middle) finger is the most affected, followed in decreasing order by the fourth (ring), fifth and then second (index) fingers. The radial or ulnar portion of a sagittal band tends to be torn, rather than in the midline component and most often the superficial fibres are involved.^5^ Sagittal band tears can be defined as partial or complete.

**Figure 10:**
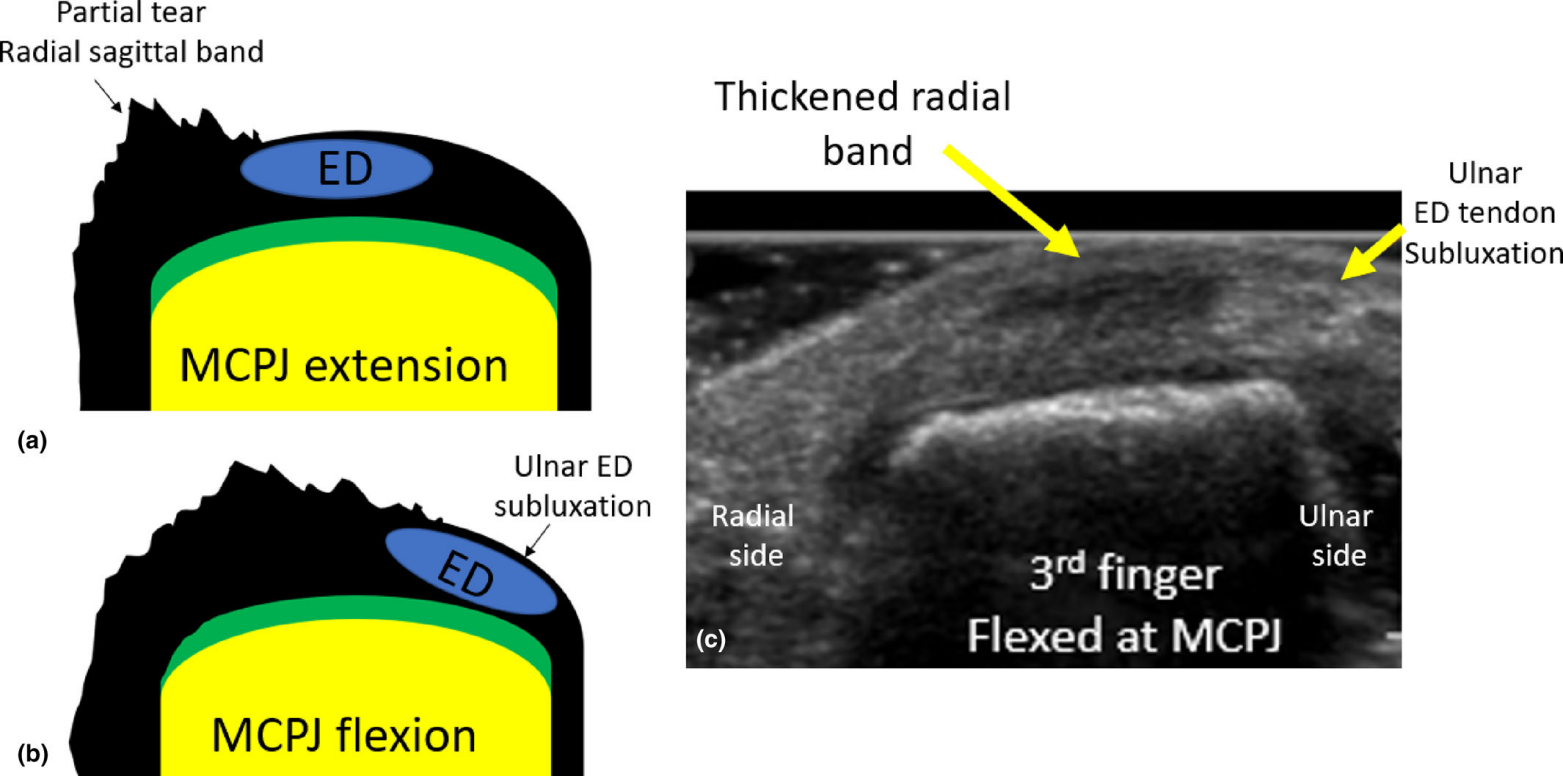
Diagram of a partial tear of a radial sagittal band at the third MCPJ with the MCPJ extended (a) and flexed (b). The ED tendon subluxates to the ulnar dorsal aspect of the metacarpal head with MCPJ flexion. (c) Short‐axis sonographic image of the dorsal aspect of the third MCPJ. The radial sagittal band appears thickened and hypoechoic and irregular in outline. There is ulnar subluxation of the ED tendon with MCPJ flexion, and the ED tendon still maintains contact with the dorsal aspect of the metacarpal head but has moved to the ulnar dorsal aspect of midline. ED, extensor digitorum; MC, metacarpal; MCPJ, metacarpophalyngeal joint; PP, palmar plate.

Partial sagittal band tears sonographically demonstrate a focally thickened and hypoechoic sagittal band on either the radial or ulnar side.^17^ A partially torn sagittal band does not show a complete gap between band ends, and the extensor tendon/s remains encapsulated by the sagittal band when the MCPJ is flexed and extended. Partial sagittal band tears can result in extensor tendon subluxation. Partial tears through 50% of the depth of the proximal radial sagittal band have been demonstrated to be sufficient to cause extensor subluxation; however, partial tears of the distal sagittal band are most often not associated with extensor tendon subluxation.^10^ In digits 3 and 4, ED tendon subluxation at the MCPJ occurs when the ED tendon moves to either the ulnar or radial sides of the midline but remains in contact with the dorsal aspect of the MC head during MCPJ flexion. The tendon subluxates to the opposite side of the partial tear, due to force applied to the central tendon by the uninjured sagittal band.^11^ Subluxation is most obvious with MCPJ flexion (forming a fist).^22^ For example, if there is a radial‐sided sagittal band partial tear, the ED tendon will subluxate to the ulnar side (Figure 10).

Complete sagittal band tears (ruptures) demonstrate a gap between radial or ulnar aspects of a sagittal band with short‐axis sonographic imaging. This results in lack of continuity of the sagittal band surrounding the ED tendon at the MCPJ level. Complete sagittal band tears can result in extensor tendon subluxation or dislocation, and the extent of tendon displacement and distinction between tendon subluxation and dislocation must be appreciated. The gap in the sagittal band and subsequent tendon instability may not be obvious with static imaging with the MCPJ in extension, so dynamic sonographic assessment with the MCPJ in multiple degrees of flexion is required.

**Figure 11:**
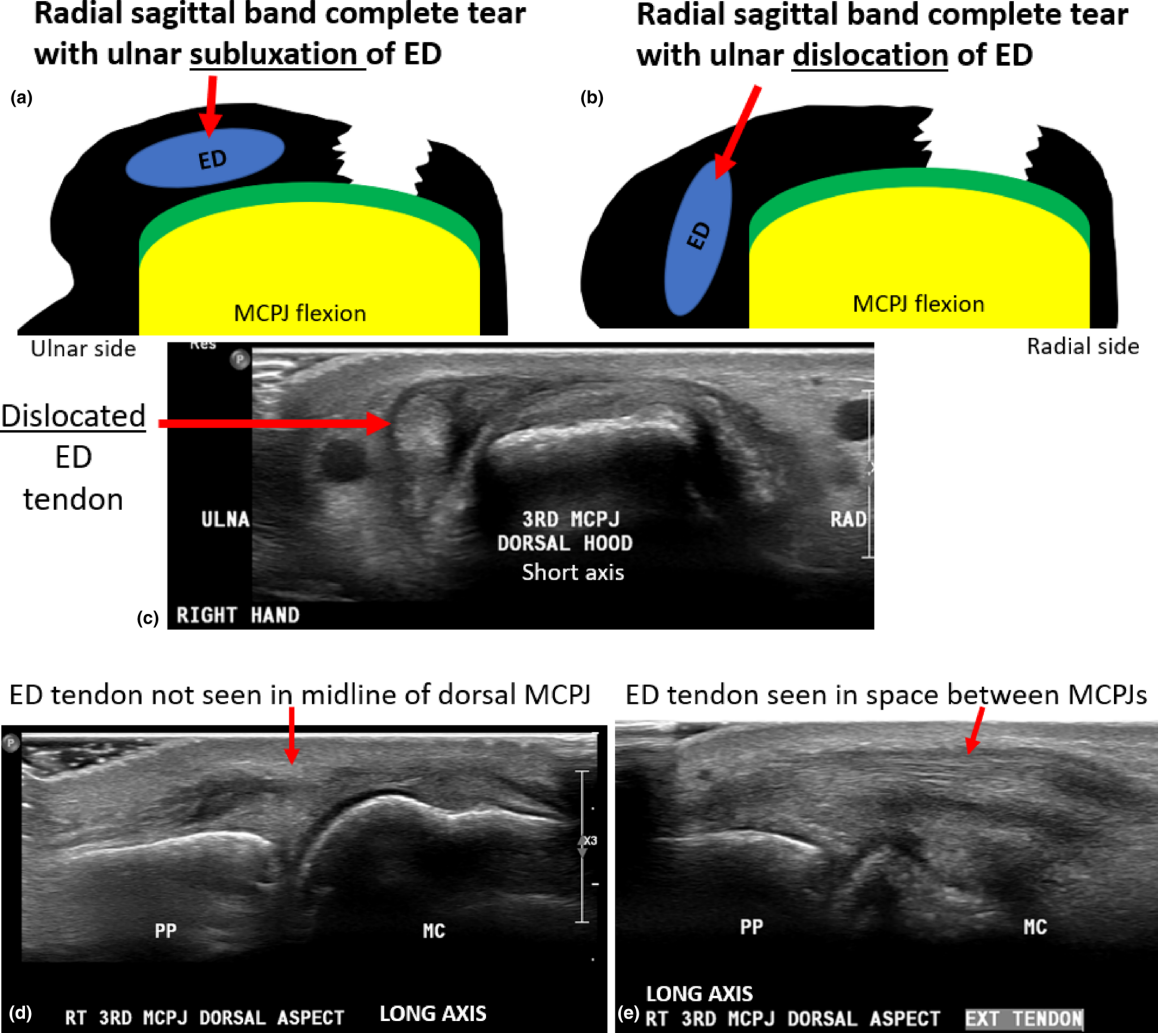
Complete tear of the radial aspect of the sagittal band. (a) Complete tear of the radial sagittal band with ulnar subluxation of the ED tendon. The ED maintains contact with the dorsal aspect of the MC head (but deviates from the ulnar dorsal aspect of midline) during MCPJ flexion. (b) Complete tear of the radial sagittal band with ulnar dislocation of the ED tendon with MCPJ flexion. When dislocated, the tendon moves into the space between MC heads and is no longer in contact with the dorsal aspect of its corresponding MC head. (c,d) Short‐axis sonographic image of the dorsal aspect of the third MCPJ. The EDC tendon is dislocated and is displaced to the ulnar aspect of the metacarpal head due to the complete tear of the radial sagittal band. (d,e) Long‐axis images over the midline dorsal aspect of the MCPJ (d) where the ED tendon is not visualised in the midline. (e) Long‐axis image acquired in the space between dorsal MCPJs where the subluxated ED tendon (subluxated in the MCPJ extension) can be seen in long axis. ED, extensor digitorum; MCPJ, metacarpophalyngeal joint; PP, proximal phalanx; MC, metacarpal; RAD, radial aspect of short‐axis image; ULNA, ulnar aspect of short‐axis image.

Transient subluxation of the extensor tendon with flexion involves maintenance of contact of the tendon with the dorsal metacarpal condyle. Dislocation of extensor tendons involves displacement of the tendon into the groove between adjacent dorsal MC heads (valley between adjacent knuckles) and loss of contact with the dorsal aspect of the metacarpal head.^16^, ^32^ In complete sagittal band tears of the third and fourth MCPJs, ED tendon dislocation occurs when the tendon moves to the opposite side of the MC head relative to the side of the sagittal band tear. Subluxation or dislocation is best demonstrated when the finger of interest is flexed to touch the palm of the hand (Figure 11 and Video 1).

**Video Video 1:** Transcription boxer's knuckle video (29 s). To dynamically assess the sagittal bands, you need to ensure that you perform flexion of the metacarpophalangeal joints (MCPJ), and the fingers must be brought all the way down to the palm. This will allow us to demonstrate some real‐time movement which can demonstrate in this case dislocation of the extensor digitorum tendon, which is dislocating to the ulnar aspect of the dorsal aspect of the MCPJ.

When complete sagittal band tears occur to the second and fifth MCPJs, due to the presence of multiple tendons, ruptures of the connections between these tendons have been identified to also occur and one of the extensor tendons may displace to the radial side and one to the ulnar side of the MC head relative to midline.^19^ The radial sagittal band is reported to be more susceptible to injury; this theory has been proposed as the radial sagittal band has been identified to be thinner and longer than the ulnar component on cadaveric studies.^33^, ^34^ Ulnar‐sided sagittal band tears although not as common, are still encountered, and traumatic lacerations can be a cause.^6^, ^11^

Radial subluxation of the ED tendon may occur following a traumatic laceration to the ulnar sagittal band.^16^ Complete tearing of the ulnar sagittal band doesn't contribute to the same degree of extensor instability with MCPJ flexion or extension as tears of the radial sagittal band, which has been attributed to the juncturae tendini.^5^ Sagittal bands may also become torn in repetitive injuries and conditions such as rheumatoid arthritis where it is associated with chronic synovitis.^16^ In arthritic patients, the superficial layer of the sagittal bands has been reported to rupture spontaneously from light, normal daily activity such as snapping, crossing a finger or crumpling paper.^14^

The extensor tendons may be concurrently partially torn in association with a sagittal band tear. Partially torn extensor tendons may sonographically appear increased in thickness and decreased in echogenicity in comparison with the contralateral asymptomatic limb. In addition, disrupttion to the fibrillar echotexture will be identified. Trauma to the sagittal bands may also result in a concurrent structural injury involving the MCPJ capsule, juncturae tendinum, palmar plate, intrinsic muscles of the intermetacarpal spaces and osteochondral fractures.^5^ The dorsum of the MCPJ can also be infected *via* a puncture wound that occurs when the blow occurs to the open mouth with a clenched fist.^13^ This is known as ‘fight bite’. The bite can cause tears to the sagittal bands and extensor tendons, and the wound can cause infection that can extend deeper to involve the MCPJ and bones.^22^

Plain hand radiographs following trauma to the dorsum of the hand are required to exclude or identify any fractures. Magnetic resonance imaging (MRI) can be utilised to image structures of the hand including the collateral ligaments of the MCPJs of digits 2–5 which, due to their position between the MC heads can be better imaged with MRI. The extensor hood of the hand may require MRI sequences to be obtained with the MCPJ in the maximum flexion and extension to demonstrate any dislocation or subluxation of the extensor tendons. Ultrasound imaging has the advantage of being dynamic and quick and can be performed in an emergency setting. Direct transducer pressure over the torn sagittal bands in the acute setting, however, can cause some patient discomfort. As ultrasound is an operator‐dependent imaging modality, knowledge of the anatomy, mechanisms of injury and sonographic technique is also required to allow the structures of the dorsal hand to be optimally imaged.

Different methods of treatment of sagittal band tears include conservative management or surgical repair, and optimal management of sagittal band tears remain undefined.^5^ The main aim is to prevent the re‐dislocation of extensor tendons and maintain the MCPJ motion. Conservative management involves the use of extension splinting.^5^ Numerous surgical techniques have been described but mostly involve relocation of the central tendon, and direct repair of the sagittal band defect with sutures.^5^

## Conclusion

The dorsal hood is a complex retinacular system of the hand. Injuries to the sagittal bands of the dorsal hood should be considered following blunt trauma to the dorsal hand such as boxing or punching, with subsequent pain and swelling to the dorsal knuckles and space between knuckles. The sagittal bands are the most important stabilising component of the extensor tendons and partial or complete sagittal band tears and can result in extensor tendon subluxation or dislocation, which may be clinically underappreciated. Sagittal band tears and the degree of associated tendon instability can be efficiently and effectively imaged with ultrasound; however, familiarity with the detailed relative anatomy, sonographic technique, and normal and abnormal sonographic appearances is essential to allow a timely diagnosis to optimally guide patient management.

## Conflict of Interest

No conflicts of interest to declare.
